# The clinical and immunological basis of early food introduction in food allergy prevention

**DOI:** 10.3389/falgy.2023.1111687

**Published:** 2023-01-23

**Authors:** L. Nuyttens, L. De Vlieger, M. Diels, R. Schrijvers, D. M. A. Bullens

**Affiliations:** ^1^ Allergy and Clinical Immunology Research Group, Department of Microbiology, Immunology & Transplantation, KU Leuven, Leuven, Belgium; ^2^ Department of Pediatrics, University Hospitals Leuven, Leuven, Belgium; ^3^ Department of General Internal Medicine, Division of Allergy and Clinical Immunology, University Hospitals Leuven, Leuven, Belgium

**Keywords:** IgE-mediated food allergy, prevention, pediatric, early food introduction, immunology, non-IgE mediated food allergy

## Abstract

IgE-mediated food allergy has an estimated prevalence of 6%–10% in developed countries. Allergen avoidance has long been the main focus in the prevention of food allergy and late solid food introduction after 6–12 months of age was recommended in high-risk infants. However, the rising prevalence of food allergy despite delayed exposure to allergens and the observations that IgE-mediated sensitization to food products could even occur before the introduction of solid foods resulted in a shift towards early solid food introduction as an attempt to prevent IgE-mediated food allergy. Since then, many trials focused on the clinical outcome of early allergen introduction and overall seem to point to a protective effect on the development of IgE-mediated food allergies. For non-IgE-mediated diseases of food allergy, evidence of early food introduction seems less clear. Moreover, data on the underlying immunological processes in early food introduction is lacking. The goal of this review is to summarize the available data of immunological changes in early food introduction to prevent IgE and non-IgE mediated food allergy.

## 1. Introduction

### 1.1. Background

The prevalence of IgE-mediated and non-IgE-mediated food allergies (FAs) has increased significantly over the last years. Nowadays, in developed countries, the estimated prevalence of FA in children varies between 6%–10%. Cow’s milk, hen’s egg, soy, peanut, tree nuts, wheat, fish and seafood are the eight most common IgE-mediated food allergies in the pediatric population (1–4). In the 1990’s, strategies to prevent food allergy focused on the avoidance of food allergens in high-risk infants and in their mothers during pregnancy and breastfeeding (3). These recommendations were built on the findings that the immature intestinal immune system plays a pivotal role in the failure of tolerance acquisition during neonatal life and infancy (5). Indeed, at birth, gut barrier function is immature and the neonate is thought to be prone to develop allergic diseases. However, breastmilk contains different trophic factors such as TGF-β and butyrate, known to stimulate the maturation of the gut barrier and decrease the permeability of the intestinal epithelial to intact proteins (6, 7). Maternal milk is moreover of utmost importance to stimulate the gut microbiota formation of the newborn (6). Altogether, these factors have an important impact on the maturation of the immune function and the differentiation of Treg cells, potentially leading to the prevention of food allergies (6, 7). Food protein transfer *via* breast milk is the first exposure to food products for the infant. There is, however, only limited data on maternal food consumption and the subsequent appearance of food proteins and antigens in maternal milk and studies concerning the impact of breastfeeding on the prevention of food allergies show inconsistent results. A possible explanation for these inconsistent results in literature is the maternal variation in breast milk composition and thus allergen and allergen-specific immunoglobulin content, as well as the variation in the definition of exclusive breastfeeding used in those studies (6). Randomization between breastfeeding and formula feeding is ethically not acceptable so randomized controlled trials are lacking. A Cochrane meta-analysis dating from 2012 analyzed 23 independent studies and showed no long-term impact on the development of allergic diseases in children who were exclusively breastfed for 6 months in comparison to children who were initially exclusively breastfed for 3 or 4 months and continued partial breastfeeding afterwards (8). Although the impact of breastfeeding on food allergy prevention remains unclear, the World Health Organization (WHO) advices to exclusively breastfeed all infants until the age of 6 months and to start adequate complementary foods as of 6 months of age while continuing to breastfeed for up to 2 years, given the benefits of breastfeeding such as immune mediators and trophic factors besides its nutritional quality (9). On top of breast milk, timing of weaning has become more and more relevant in food allergy prevention. Studies showed the importance of early introduction of allergens in children at high risk of developing IgE-mediated food allergy. At present, the exact timing of solid food introduction is of great interest to prevent the development of early onset food allergy and more clinical data has become available over the last years. However, immunological data is still largely lacking and the humoral and cellular basis of early allergen introduction remains mostly unknown (10). The goal of this review is to summarize the findings of the immunological changes in early food introduction to prevent IgE and non-IgE-mediated food allergies. First, we will describe the most important findings of clinical research in early antigen introduction in the prevention of IgE-mediated food allergy. Second, we will discuss the immunological changes accompanying the development of tolerance to these food allergens. At last, we will focus on the immunological pathophysiology of non-IgE-mediated FAs, as literature in this field is scarce.

### 1.2. Shift from allergen avoidance guidelines to early introduction in allergy prevention

The avoidance of potential allergens has long been the main goal in primary (the prevention of the development of allergy), secondary (the prevention of disease progression) and tertiary (the prevention of symptoms in allergic subjects) prevention of allergic diseases. While allergen avoidance is still the gold standard for the prevention of allergic reactions, its role in primary prevention is debated (11).

Over the last decades, there have been important changes in recommendations concerning the timing of the introduction of solid foods to infants (11, 12). In the 1990’s allergen avoidance and late solid food introduction after 6 to 12 months of age was recommended in high-risk infants (i.e., familial predisposition, eczema) (3, 11, 12). This advice was based on the hypothesis of immaturity of the mucosal immunity during childhood, making sensitization towards food antigens easier (3). However, the rising prevalence of FA despite delayed exposure to allergens had led to a reconsideration of these recommendations (12). Since then, the WHO advocates to introduce solid foods to all infants from 6 months onwards in complementation of breast milk until 2 years of age (3, 9). However, this advice was not formulated for the prevention of food allergies, but merely to improve the benefits and protective effects of maternal milk against gastrointestinal and respiratory infections, as this still represents an important cause of mortality in developing countries (12). Moreover, several studies show early sensitization to food products, even before the introduction of solid foods. For example, the “Starting Time for Egg Protein” (STEP) trial was designed to study whether frequent consumption of hen’s egg from age 4–6 months reduces the risk of IgE-mediated hen’s egg allergy in infants with an atopic mother. In this study, 5% of the high-risk infants at randomization in 2010–2012 were sensitized to hen’s egg before any known ingestion of hen’s egg in solid foods (13). Also, the “Preventing Atopic Dermatitis and Allergies in children” (PreventADALL) study aimed to examine the effect of the regular use of skin emollients from the age of 2 weeks on and/or early complementary food introduction between the age of 12 and 16 weeks, on the development of atopic dermatitis at the age of 12 months and the sensitization status at the age of 3 months. This PreventADALL study showed that 7% of infants from a general population in 2015 to 2017 were sensitized to food allergens at the age of 3 months, thus before the introduction of solid foods (14–16). Therefore, earlier preventive measures seem necessary, although sensitization after this period can also certainly occur. The early introduction of allergens before 6 months of age is hypothesized to reduce the development of FA but seems to have no influence on the total duration of breastfeeding, supporting the evidence that early introduction of solids could coexist with the continuation of breastfeeding. Early food introduction might therefore not be in contrast to WHO recommendations (12). Furthermore, the European Academy of Allergy and Clinical Immunology (EAACI) advices against the avoidance of food allergens during pregnancy or breastfeeding. Also, EAACI counter advices cow’s milk formula as supplementary feeding for breastfed newborns in the first week of life or exclusive soy protein formula in the first 6 months of life to prevent FA. However, they recommend to all newborns the introduction of cooked egg from 4 to 6 months of life, as well as the introduction of peanut in the same window period in populations where there is a high prevalence of peanut allergy (17).

## 2. Methods

For this review, we examined available literature in Pubmed with one or with a combination of the following keywords: IgE-mediated food allergy, non-IgE-mediated food allergy, prevention, early food introduction, pediatric, tolerance, immunology. We included randomized controlled trials, reviews and observational prospective and retrospective studies from January 1, 2013 to June 1, 2022. For the topic of non-IgE-mediated food allergies, case reports were also included as literature was limited. Only English written publications with full text availability were retained, abstracts were excluded.

## 3. Clinical outcome of early solid food introduction

First, an important role of dietary factors in the development of allergies has been described. Roduit *et al*. studied the impact of a low food diversity score in the first year of life on the development of allergic diseases such as atopic dermatitis and food allergies. Food diversity score was based on the count of the different complementary food products introduced before the age of 1 year. These food products consisted out of the following allergenic items: cow’s milk, soy, hen’s eggs, nuts, vegetables or fruits, cereals, bread, meat and fish. A higher food diversity score (i.e., more different food products introduced before the age of 1 year) was inversely associated with the development of food allergy up to the age of 6 years and with sensitization to food allergens (IgE cutoff 3.5 kUA/L) at the age of 4.5 and 6 years (18). A follow-up study of the same study group showed that the continued consumption of dairy products and hen’s eggs within the second year of life was associated with a lower risk to develop allergic diseases (asthma, food allergy, atopic dermatitis) by 6 years of age (19). Although, these results can be compatible with a protective effect of early milk and hen’s egg consumption, children who avoid these food products might as well be early allergic and prone to start “the Atopic March”. Early allergy diagnosis might therefore be of utmost importance (20).

The skin prick test (SPT) is widely used as an objective diagnostic tool for IgE-mediated food sensitization in clinical settings (21, 22). SPT observations in early peanut introduction studies showed small wheal size in the early consumption group without prior peanut sensitization (SPT < 4 mm), whereas an increase in wheal size compared to baseline was seen in the avoidance group (23, 24). Furthermore, the “Enquiring about tolerance” (EAT) trial showed a 22% lower risk of a positive SPT to milk, peanut, hen’s egg, sesame, fish or wheat in the early introduction group vs. the standard introduction group at 12 months of age and a 12% lower risk at 36 months of age in the per protocol analysis. However, only 63% of the children in the total study population and hardly 37% of the children randomized in the early introduction group were able to adhere to the protocol (25).

The introduction of solid allergenic foods before the age of 6 months indeed seems overall beneficial to reduce the risk of IgE-mediated FA. An overview of published clinical studies concerning early solid food introduction since 2013 can be found in Supplementary Table S1 (13–16, 23–34).

## 4. Immunological changes in early solid food introduction in the prevention of food allergy

### 4.1. Prenatal start of tolerance induction

The development of FA can be influenced by genetic, epigenetic and environmental factors. There are hundreds of genes involved in the development of IgE-mediated FA. Environmental and epigenetic factors like DNA methylation, histone modification and microRNA’s can alter the expression of these genes. Studies regarding DNA methylation changes in cytokine genes such as interferon gamma (IFN-γ), interleukin-4 (IL-4) and IL-5 and in Forkhead box P3 (FoxP3) indicate that changes in DNA methylation may predispose IgE-mediated food sensitization or allergy in early childhood (35–38). Acevedo et al. studied the DNA methylation levels in mononuclear leukocytes from mothers and their children. In this study peripheral blood mononuclear cells (PBMC’s) were collected from mothers during pregnancy and from the offspring at birth *via* cord blood and at the age of 2 and 5 years. DNA methylation levels were compared at each timepoint between children with and without IgE sensitization to different allergens at the age of 5 years. Multiple differently methylated regions associated with IgE sensitization to food and aero-allergens were described. Furthermore, maternal DNA methylation status was associated with IgE sensitization in the offspring supporting early *in utero* effects on atopy predisposition (37). Also, Han et al. showed that DNA hypermethylation at 5′ cytosine-phosphate-guanine-3′ (CpG) sites at birth, measured in cord blood or Guthrie cards, was associated with higher IgE levels later on in childhood (38). Secondly, histone modification by phosphorylation, acetylation, methylation or ubiquitination alter DNA accessibility and leads to decompression or compression of chromatin which influences gene expression. Lastly, microRNAs are small non-coding RNAs that can modify post-transcriptional gene regulation *via* interaction with target mRNA (35, 36).

These studies support the hypothesis that early sensitization, even *in utero,* can occur (30, 37, 39, 40). The FONIA study of Bullens et al. showed that sensitization as early as at birth (total IgE levels of ≥0.35 kU/L) was significantly associated with the development of early allergy before the age of 2 years. Furthermore, FoxP3/CD3γ mRNA ratios were compared between healthy and allergic subjects. This ratio can be used to represent the fraction of regulatory T (Treg) cells amongst the T cell population. FoxP3/CD3γ mRNA ratios in cord blood were significantly lower in early allergic children than in healthy children. Late allergic children (who develop allergy between the ages of 2 and 6 years) however had similar cord blood FoxP3/CD3γ mRNA ratios as healthy children (39). Likewise, Prince et al. also described differences in Treg frequency associated with age. Young food allergic children (0–6 years of age) had significantly lower percentages of peripheral blood CD4^+^CD25^hi^CD127^lo^Foxp3^+^ Treg cells in comparison to healthy controls of the same age (41). These data indicate that postnatal factors can also certainly influence allergy development.

### 4.2. Postnatal changes during tolerance induction

#### 4.2.1. Environmental factors

As described above, environmental factors and epigenetic factors can alter gene expression leading to the development of FA. Vitamin D deficiency, for example, has been shown to predispose to the development of FA by alterations in DNA methylation of different genes associated with FA (35, 36). However, the role of vitamin D is still under debate as high levels of vitamin D have also been described to suppress Treg frequency through the methylation of FoxP3, leading to the development of FA (42). Also, methyl group donors, such as folic acid can predispose to allergic disease by the alteration of methylation patterns. Furthermore, butyrate, a short-chain fatty acid, has been described in the prevention of FA. It plays an important role in the absorption of electrolytes by the intestine, in the mucosal integrity and in local and systemic metabolic function (35, 36). Therefore, butyrate has a significant effect on the gut microbiome leading to the stimulation of regulatory immune responses and the gut microbiome itself also has an important effect on allergy development, as reviewed by Chernikova and colleagues (43).

#### 4.2.2. IgE, IgG and IgG4 production and B-cell responses

In order to define the underlying immunological events associated with tolerance induction in relation to early food introduction, sensitization to food allergens as outcome measure has often been studied. Although the expectation was that food specific IgE (sIgE) levels might not be present in tolerant children, this was not necessarily the case. Indeed, as seen in oral immunotherapy (IT) trials for food allergy, during which a food allergic patient ingests increasing doses of their allergen to desensitize their immune system, the presence of oral tolerance during early solid food introduction is not necessarily associated with the absence of allergen-sIgE (21, 44). In allergen-specific IT, an early boost in allergen-specific B cell activation is seen. During the following months, this initial boost is followed by a reduction in allergen-sIgE and an increase in allergen-sIgG4 (21). Similarly in early food introduction, hen’s egg-sIgE levels in the STEP study were higher in the hen’s egg consumption group in comparison to the hen’s egg avoidance (control) group at age 12 months (13). Nishimura et al. also showed an increase in sIgE for egg white, milk, wheat, soybeans, buckwheat and peanut from baseline to 18 months in an early food introduction group and only the median egg white sIgE levels were lower in the intervention group than the placebo group (34). In contrast to this, the “Prevention of egg allergy with tiny amount intake trial” (PETIT study) showed a lower ovomucoid-sIgE concentration at 12 months of age in the hen’s egg group compared to the hen’s egg avoidance group (31). Similarly in the “Learning early about peanut allergy” (LEAP) study, the number of subjects with markedly elevated levels of peanut-sIgE titers at 60 months was lower in the peanut consumption than in the avoidance group (23).

In line with the latter, Roduit et al. studied whether food diversity in the first year of life had an impact on the expression of Cɛ germline transcripts in B-cells at the age of 6 years. The Cɛ germline transcript is a marker for antibody isotype switching to IgE and its inhibition seems to play a role in the inhibition of allergy development by Treg cells. Roduit et al. showed that among children with limited food diversity within the first year of life, the expression of the Cɛ germline transcript in B-cells at the age of 6 years is significantly increased in comparison to children with a high food diversity (18). Consequently, B cells will express IgE with various effector functions, but with retained antigen specificity, resulting in broader sensitization patterns and/or food allergy (45). Hence, early food introduction will probably lead to decreased IgE antibody isotype switching.

**Figure 1:**
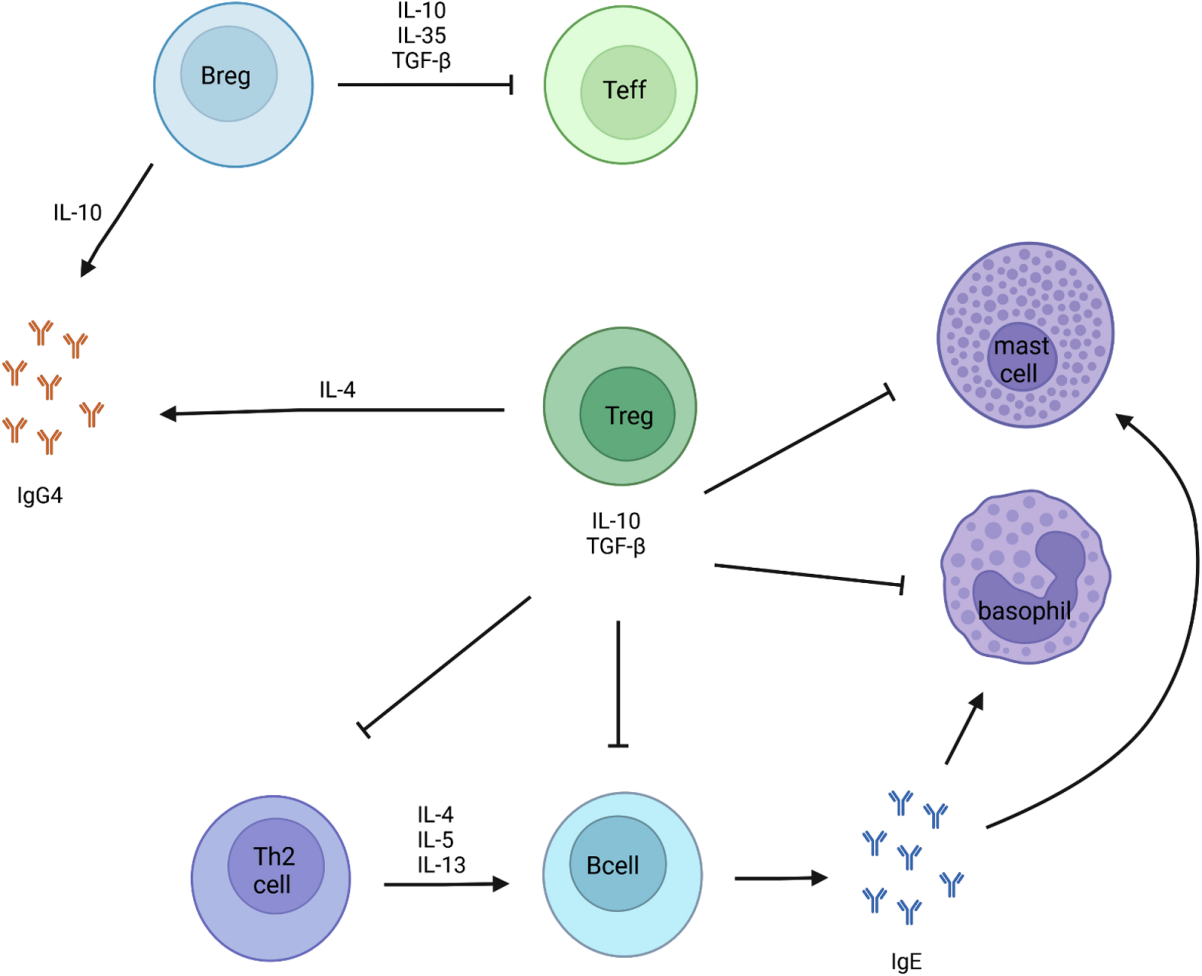
Markers of tolerance development to food allergens. IL-10 secreting Breg cells, are known for being the main producers of IgG4. Through the secretion of IL-10, IL-35 and TGF-β, Breg cells suppress the activation and proliferation of effector T cells. Regulatory T cells inhibit mast cells, basophils, Th2 cells and B cells *via* the secretion of IL-10 and TGF-β.

In contrast to the results related to allergen sIgE, allergen-specific IgG4 (sIgG4) is more consistently associated with oral tolerance (44). Allergen sIgG4 antibodies can compete with IgE for allergen binding to type II IgE receptor-expressing cells, such as basophils and mast cells leading to the blockage of this receptor. The production of both IgE and IgG4 antibodies is known to be up-regulated by IL-4 produced from activated Th2 cells. However, IL-10 secreted by Treg cells can suppress IgE production and simultaneously increase IgG4 production (Figure 1) (46, 47). Therefore, IgE and IgG4 production are not always similarly increased.

In the LEAP study, the peanut consumption group showed a significantly greater and earlier increase in peanut specific IgG and IgG4 levels in comparison to the avoidance group. Furthermore, in the avoidance group, elevated peanut sIgG4 levels were associated with the absence of an allergic reaction to peanut upon consumption. These findings indicate that IgG4 could be associated with a protective role against allergy development (23). Likewise for hen’s egg, the “Solid timing for allergy research” (STAR) study showed a significant increase in hen’s egg specific IgG4 levels and IgG4/IgE ratios in infants with moderate to severe eczema who underwent early hen’s egg introduction (32). Also, the “Beating egg allergy” (BEAT) study demonstrated a similar response in infants with absent or mild eczema and extended the observations to the major hen’s egg protein components ovomucoid and ovalbumin at 12 months of age (28). The PETIT study showed a higher ovomucoid-specific IgG1, IgG4 and IgA concentration at 12 months of age in the hen’s egg consuming group than in the placebo group (31) and in the STEP study early ingestion of hen’s egg was also associated with higher egg-specific IgG4 levels at age 12 months (5).

Additionally, it seems that the timing of the effect of early food introduction on IgG4 levels is sooner detectable in comparison to the IgE levels. In the LEAP study for example, peanut-specific immunoglobulins were measured in serum of participants in the peanut-consumption group at baseline (between 4 and 11 months of age) and at 12, 30 and 60 months of age. Whereas peanut-specific IgG4 levels were elevated as early as month 12, a significant decrease in the mean levels of Ara h2-sIgE had only occurred by month 60 in this peanut-consumption population (24). This can possibly be explained by the initial rise in IgE after exposure to the allergen (21).

Not only are IgE and IgG4 important to predict reactivity to an allergen or to confirm food tolerance, also the ratio of IgE to IgG4 seems to play a key role (40, 46). Okamoto et al. studied the concentrations of hen’s egg sIgE and IgG4 antibodies during oral food challenges (OFC) with egg white in children sensitized to hen’s egg. During OFCs, gradually increasing amounts of the food to be tested, are eaten and symptom occurrence is observed under medical supervision. It remains the gold standard to accurately diagnose or rule out an allergy to the tested food. In the study of Okamoto et al., hen’s egg sIgE concentrations were higher in children with a positive OFC than in children with a negative OFC. On the other hand, the concentration of hen’s egg sIgG4 antibodies was higher in the negative OFC than in the positive food challenge. However, the ratio of IgE to IgG4 seemed to be an even more useful parameter than IgE or IgG4 alone to predict clinical reactivity during hen’s egg challenge as it also had a more accurate sensitivity and specificity (46, 47). Similarly for cow’s milk, Savilahti et al. showed higher levels of cow’s milk sIgE in cow’s milk allergic children compared with those who became tolerant by the age of 3 years, but cow’s milk sIgG4 serum concentrations were lower among patients with persistent cow’s milk allergy than among patients tolerating cow’s milk by the age of 3 years (47, 48).

Regulatory B (Breg) cells are immunosuppressive B cells, characterized by the production of IL-10, IL-35 and transforming growth factor beta (TGF-β). IL-10 secreting Breg (Br1) cells, are known for being the main producers of IgG4 (49). Through the secretion of IL-10, IL-35 and TGF-β, Breg cells are known to suppress the activation and proliferation of effector T cells, and also induce the differentiation of FoxP3^+^ regulatory T cells and type 1 regulatory (Tr1) cells (Figure 1). In the context of early food introduction, Lai et al. showed that the development of hen’s egg allergy during infancy was accompanied by a reduction in the frequency of ovalbumin-specific Breg cells, consistent with defective tolerance induction. They described a significant lower ovalbumin-specific IL-10^+^ CD19^+^ B cells and CD19^+^CD25^+^CD71^+^ Breg cells at the age of 12 months in hen’s egg allergic infants. This was not observed in non-hen’s egg sensitized or hen’s egg sensitized but tolerant infants after early hen’s egg exposure (4).

#### 4.2.3. T cell responses

**Figure 2:**
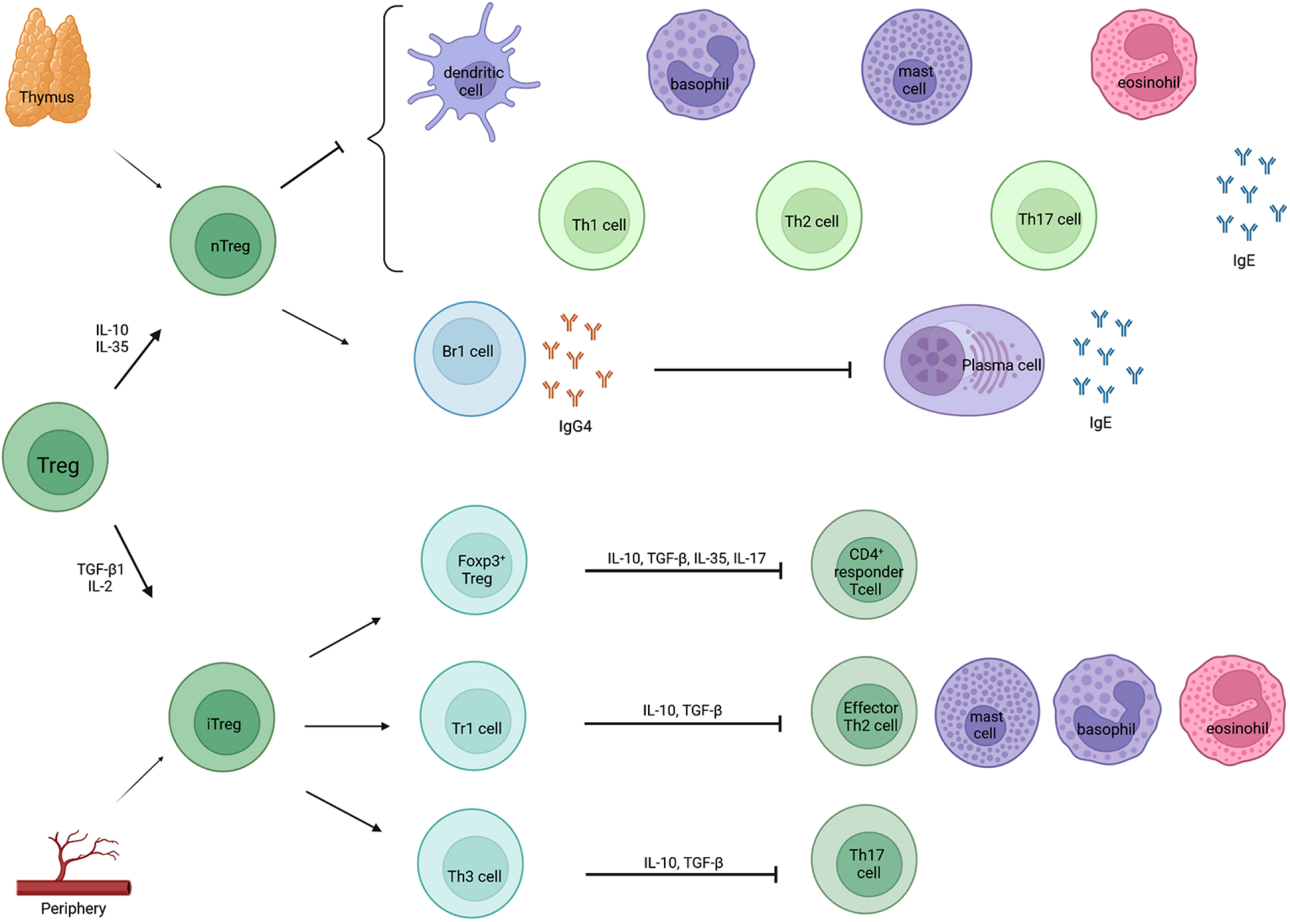
Classification and function of Treg cells. Treg cells can be classified in two categories: natural Treg cells (nTreg) and inducible Treg cells (iTreg). Natural Treg cells are produced in the thymus after contact with self-antigens, whereas iTregs are derived from the periphery after contact with foreign antigens. Natural Treg cells inhibit different effector cells and the production of allergen sIgE and they induce IgG4 by Br1 cells which further blocks IgE activity *via* the inhibition of plasma cells. Inducible Treg cells can be subdivided into three subsets: induced Foxp3^+^ Tregs, type 1 regulatory T cells (Tr1 cells) and T helper 3 cells (Th3 cells). They, in turn, inhibit CD4^+^ responder T cells, effector Th2 cells, mast cells, basophils, eosinophils and Th17 cells.

Allergic diseases are characterized by a Th2 immune response. However, Treg cells are a distinct subpopulation of CD4^+^ T cells that play an important role in the establishment and maintenance of immune homeostasis. The function of these Treg cells is to suppress the effector function of a wide range of cells and to prevent inadequate immune responses. They are categorized into natural Treg (nTreg) cells, produced in the thymus in response to self-antigens, and inducible Treg (iTreg) cells, generated in the periphery from naïve CD4^+^ T cells in response to foreign antigens (Figure 2). Natural Treg cells are identified as CD4^+^CD25^+^FoxP3^+^ cells that produce IL-10 and TGF-β. Their role in allergen-specific immune reactions include suppression of dendritic cells that support the generation of effector T cells. Furthermore, they inhibit the function and migration of effector Th1, Th2 and Th17 cells and they inhibit the production of allergen sIgE as well as induce IgG4 secretion. Furthermore, they suppress eosinophils, mast cells and basophils. Inducible Treg cells can be subdivided into three subsets: induced FoxP3^+^ Treg cells, Tr1 cells and T helper 3 cells (Th3 cells) (49). FoxP3^+^ Treg cells express CCR6 and have the capacity to produce IL-17 upon activation. They inhibit the proliferation of CD4^+^ responder T cells. Tr1 can transiently express FoxP3 upon stimulation, whereas Th3 cells do not express FoxP3 (50, 51). However, the transient expression of FoxP3 on Tr1 cells does not correlate with its suppressive function and FoxP3 independent mechanisms contribute to the suppressor functions of Tr1 cells (51). Furthermore, Tr1 cells can be distinguished by flow cytometry by the co-expression of the surface markers CD49b and LAG3 and the secretion of high levels of IL-10. They home to the lung and draining lymph nodes. In allergen-specific immune reactions, they inhibit the function and migration of effector Th2 cells and they suppress eosinophils, mast cells and basophils. Th3 on the other hand expresses TGF-β. Whether these subsets of iTreg cells are all distinct cell populations or perhaps have some overlapping stadia, is not entirely known (50).

The Treg cell count was similar or even higher in infants compared to adolescents and adults, given their involvement in maternal-fetal tolerance. The immaturity and plasticity of the immune system, together with the maintained thymic functionality in children, could be decisive in both the onset of FA and the acquisition of tolerance to allergens (49). Treg cells can control the sensitization and effector phases of allergic reactions through multiple suppression mechanisms and both Treg cell deficiency and Treg cell dysfunction have been described in the process of allergy development (49, 52). Akdis et al. showed that higher frequencies of allergen-specific Treg cells were encountered in healthy patients and that Treg cells isolated from the blood of house dust mite or birch pollen allergic patients were less functional than Treg cells isolated from healthy subjects (53). Likewise in food allergy, the state of allergen sensitization can be associated with depletion of Treg cells following hen’s egg, cow’s milk or peanut exposure and the impaired capacity to regenerate the Treg pool is an important factor to determine clinical allergy versus a sensitized but tolerant state (49, 52).

One of the first reports examining the characterization of the immunological changes associated with tolerance in early allergen exposure focused on participants of the BEAT and STEP trial. Firstly, an age-related increase in ovalbumin-specific Treg cells (CD137^+^IL-10^+^ and CD137^+^FoxP3^+^ cells) was observed in infants who consumed hen’s egg on a regular basis early in life. This increase was not observed when the total FoxP3^+^ Treg population was examined, indicating that this change was hen’s egg specific. Furthermore, an age-related increase in ovalbumin-specific Treg cells was also observed between the ages of 5 and 12 months in subjects who did not develop hen’s egg allergy. This increase could not be observed in infants who developed hen’s egg allergy. Unfortunately, due to insufficient sample size, it could not be examined in subjects sensitized but tolerant to hen’s egg (10). Likewise, Roduit and colleagues also noticed a significantly lower level of gene expression for FoxP3 at the age of 6 years in children with a low food diversity score within the first 12 months of life (18).

Similar as with hen’s egg, most of the patients with cow’s milk allergy outgrow their allergy, making this disorder interesting for studies on the development of tolerance (54). Karlsson et al. studied the T cell responses in cow’s milk allergic children who, after a milk-free period, reintroduced cow’s milk to their diet. They observed that children who outgrew their allergy, had higher frequencies of circulating CD4^+^CD25^+^ Treg cells after *in vivo* milk exposure and that these cells suppressed the effector T cell function. Furthermore, PBMC’s showed higher proliferative activity against β-lactoglobulin in patients with an active cow’s milk allergy compared to children who became tolerant for cow’s milk (54).

#### 4.2.4. Effector T cell-mediated cytokine responses

During prenatal life, there is a Th2-skewed immunity under the influence of pregnancy hormones such as progesterone, human chorionic gonadotrophin, estradiol and prostaglandin D2 (55). Allergen-specific responses have been demonstrated as early as at 22 weeks gestation and also cord blood mononuclear cells seem to be able to respond to environmental allergens (56). However, it is not clear whether these responses reflect conventional memory responses as they do not correlate well with either maternal exposure, nor with subsequent development of allergic disease or if these responses reflect a default response by thymic emigrants to first antigen encounter, which also leads to the activation of Treg cells (56, 57).

At birth, cytokine production is still dominated by Th2 cytokines and most aspects of neonatal immunity are immature including antigen presenting cells (APCs), Th1 cells, Th17 cells and Treg functioning. Postnatal microbial exposure provides an important source of immune stimulation and determines the fate of naïve CD4^+^ T cells. IL-12 advocates differentiation of Th1 cells. IL-6 and TGF-β promote differentiation of Th17 cells and IL-4 induces differentiation of Th2 cells. Furthermore, high concentrations of IL-6 and TNF-α promote degradation of Foxp3, the master transcription factor of nTreg. In the case of allergy, Il-6 and IL-1β have been shown to break allergen-specific CD4^+^ T cell tolerance (56, 58).

Early food introduction is hypothesized to influence this fate determination of naïve T cells. In the context of early solid food introduction, Metcalfe et al. compared the cytokine responses to hen’s egg proteins in infants with eczema in an early versus a delayed hen’s egg introduction group. The early intervention group received a daily dose of pasteurized raw whole egg powder from 4 to 8 months of age, followed by the introduction of cooked egg at 8 months of age after a medically supervised introduction of hard-boiled egg. The infants in the delayed introduction group did not introduce hen’s egg into their diet until the age of 8 months, after a passed medically supervised introduction of hard-boiled egg (32). This study showed the presence of baseline early Th2 responses to multiple hen’s egg proteins in a high proportion of infants with eczema by 4 months of age. This was prior to the introduction of hen’s egg in solid foods in any of these children. Furthermore, early introduction of hen’s egg from 4 months of age on was not associated with any significant effect on hen’s egg-specific IL-13, IL-5 and IL-10, IFN-γ or TNF-α responses by PBMC’s at the age of 12 months. However, high *in vitro* baseline IL-5 responses to lysozyme and IL-13 responses to both ovalbumin and lysozyme were associated with IgE-mediated hen’s egg allergy at 12 months of age. On the other hand, no negative nor positive association was found between IL-10, IFN- γ and TNF-α responses at 4 months of age in children with IgE-mediated hen’s egg allergy at age 12 months (59). Those cytokines could however be related to tolerance induction to aero-allergens, which might differ from food allergens (44).

A study of Neeland et al. examined the innate immune profiles associated with persistent hen’s egg allergy in comparison to children who had no hen’s egg allergy or to children who became tolerant to hen’s egg. In children with hen’s egg allergy at 1 year of age (baseline), they found an increased number of circulating dendritic cells and monocytes that produce more inflammatory cytokines (IL-8 in unstimulated media, and TNF-α, IL-6, IL-1β, IL-8 and IL-10 in LPS-stimulated cells), compared to non-allergic infants (60). In transient hen’s egg allergy (i.e., children who outgrow their allergy), there is an increase in the frequency of dendritic cells during follow-up but no significant increase was seen in children with persistent hen’s egg allergy during follow-up. Furthermore, children with persistent hen’s egg allergy continued to show higher levels of IL-6, IL-1β and IL-8 in unstimulated CD3-depleted cells compared to children who developed tolerance to hen’s egg at the end of follow-up. Following LPS stimulation, IL-6 and IL-1β production was more elevated in both persistent and transient hen’s egg allergy in comparison to healthy controls at follow-up but increased production of TNF-α and IL-8 was unique to infants with persistent hen’s egg allergy (60). These data indicate that in persistent food allergy increased inflammatory responses by APCs might be observed. If those could be silenced, tolerance induction could perhaps be achieved in this group as well.

#### 4.2.5. T-cell attractant chemokine secretion

Thymus and Activation-Regulated Chemokine (TARC), also named CCL17 (CC chemokine ligand 17), is a CC-chemokine that is mostly known as the key regulator of Th2-mediated inflammation in allergic asthma and atopic dermatitis. It is synthesized by different cell types such as monocytes, dendritic cells, macrophages, eosinophils, keratinocytes, T cells and bronchial, alveolar and nasal epithelial cells. It binds to the C-C chemokine receptor type 4 (CCR4) expressed by CD4^+^ T-cells (61–63). In food allergy, TARC is not well studied. The PETIT trial of 2016 showed higher circulating TARC concentrations in the placebo group in comparison to the hen’s egg introduction group at baseline, without reaching significance. TARC concentration at 9 and 12 months of age seemed decreased from baseline in both groups (31). However, Nishumara et al. observed in 2022 similar TARC values at the age of 3–4 months (baseline) in the intervention (i.e., the introduction to mixed allergenic food powder containing egg, wheat, milk, soybean, peanuts and buckwheat during 12 weeks at the age of 3–4 months) and the placebo group (i.e., placebo powder during 12 weeks at the age of 3–4 months). Furthermore, at age 11–13 months, TARC concentrations were slightly higher in the placebo group than in the intervention group (34), suggesting more TARC and hence more Th2 orientation in late solid food introduction.

## 5. Immunological basis of non-IgE-mediated food allergy

In contrast to IgE-mediated food allergy, information regarding the immunological basis of non-IgE-mediated food allergy is scarcer in literature. Non-IgE-mediated food allergies can be subdivided into different entities: food protein-induced enterocolitis syndrome or FPIES, food protein-induced allergic proctocolitis or FPIAP, food protein-induced enterophathy or FPE, food protein-induced motility disorders (FPIMD) such as gastro-esophageal reflux disease (GERD), colic and constipation, and eosinophilic gastrointestinal disorders (EGIDs) such as eosinophilic esophagitis (EoE), eosinophilic gastroenteritis (EGE), eosinophilic enteritis and eosinophilic colitis (EC). The immunological pathophysiology varies depending on the subtype.

### 5.1. FPIES

FPIES is considered to be a non-IgE-mediated disorder, however in up to 30% of patients with FPIES low positive sIgE’s to food allergens are present (64). FPIES is a T-cell mediated disease in which antigen-specific T cells release high levels of TNF-α that acts synergistically with IFN-γ to increase intestinal permeability (64–66). This contributes to the influx of antigens into the submucosa which in turn activates antigen-specific T cells. Furthermore, TGF-β1 receptor in the intestinal epithelium is diminished leading to a cascade of inflammatory factors that weaken the integrity of the intestinal epithelial barrier (64, 65). Additionally, the cytokines IL-2 and IL-5 and chemokine IL-8 seem to be increased in serum of patients with FPIES to cow’s milk after a failed OFC with cow’s milk formula. Also, the innate immune system is involved in FPIES reactions and a dominant activation of monocytes in addition to natural killer cells, neutrophils and eosinophils is described (64).

Clinically findings suggest that most infants with FPIES outgrow their allergy over time and become tolerant to the culprit food. However, long-lasting FPIES has been described and is especially associated with the presence of sIgE towards the trigger foods (64–66). A case report of a 9-year old boy suffering from long-lasting hen’s egg FPIES was published in 2021. In this case report, oral desensitization to induce tolerance was performed and after 13.5 months, the boy could ingest a whole raw hen’s egg without adverse reactions. However, no data was collected in this case report to evaluate the immunologic process of tolerance development (67). Nonetheless, Karlsson et al. described a higher frequency of circulating antigen-specific CD4^+^CD25^+^ Treg cells in children with non-IgE-mediated hypersensitivity to cow’s milk who developed tolerance in comparison to children with active non-IgE-mediated hypersensitivity to cow’s milk (65, 66). Furthermore, another case report showed an increase in serum IL-10 levels after a negative rice OFC in comparison to IL-10 levels before the OFC in an 8-month old infant who had an allergic reaction to rice 6 months prior to the food challenge (68).

### 5.2. FPIAP, FPE and EGIDs

The immunological characteristics of FPIAP, FPE and EGIDs are even less known and data concerning the immunological changes accompanying tolerance acquisition is lacking. Similar to FPIES, TNF-α is highly secreted in FPIAP whereas TGF- β ligand and TGF-β1 receptor activity are decreased, leading to weakening of the epithelial barrier of the intestinal mucosa and promoting fluid shifts resulting in diarrhea. It is a non-IgE mediated disease, but patients with FPIAP have a two-fold risk of developing IgE-mediated food allergy. FPE on the other hand involves eosinophils, allergen specific T lymphocytes and specific cytokine profiles such as IL-4 and IFN-γ. The immunological pathogenesis of the EGIDs is poorly understood. In EoE it is known that single nucleotide polymorphisms (SNPs) in genes encoding for thymic stromal lymphopoietin (TSLP), calpain 14 protease (CAPN14), IL-1 family genes, serine peptidase inhibitors (SERPINs), serine protease inhibitors or Kazal-type-related inhibitors (SPINKs) are associated with an increased risk of EoE (64, 69). Furthermore, a localized Th2 inflammation plays an important role in the generation of the proinflammatory cytokine responses of IL-5 and IL-13. These cytokines induce the secretion of eotaxin-3 from epithelial cells, leading to the attraction of eosinophils to the esophagus. Furthermore, IL-13 can also reduce the expression levels of filaggrin, weakening the barrier function of the esophageal epithelium (64, 69). Tolerance induction in EoE does probably not occur. Early food introduction is most likely not related to EoE prevention. On the contrary, some EoE cases in oral IT trials for hen’s egg and/or cow’s milk have been described (70).

### 5.3. FPIMD

FPIMDs are mostly reported in non-IgE-mediated cow’s milk allergy, but can also occur with other food allergens, such as egg, wheat and soy (71). Similarly as for the other non-IgE-mediated entities, the pathophysiology of FPIMD remains poorly understood. The gastrointestinal motility is controlled by the enteric nervous system and there seems to be an interaction between different immune mediators such as mast cells, eosinophils and lymphocytes and this nervous system. When an allergic reaction occurs, the release of cytokines can affect the nervous system and cause motility dysfunction leading to constipation, colic or GERD (72–74). Immunological data concerning tolerance acquisition has also not yet been studied in FPIMD.

## 6. Discussion

This review shows an important shift in the clinical approach to prevent the development of documented IgE-mediated food allergies. Early food introduction seems to have beneficial effects in the prevention of these food allergies. However immunological data that supports this shift in clinical decision making is scarce and the processes of tolerance acquisition remain poorly understood. Humoral, cellular and chemokine responses all seem to play an important role in the immune mechanisms involved in tolerance acquisition.

More studies are necessary to further investigate the immunological basis of food allergy and tolerance development. Such studies however, often require repeated sampling of larger blood volumes which is often difficult to obtain in young children with only a limited circulating blood volume. Nonetheless, new techniques have become available requiring very small volumes of blood, for example the finger-prick tests, which could make the assessment of the immunological changes in early food introduction more feasible in the future.

In conclusion, early antigen introduction seems to have a protective effect on the development of food allergies. However, immunological evidence is necessary to completely understand the processes of tolerance acquisition in early food introduction in the prevention of IgE- and certainly non-IgE-mediated food allergy.

## Author contributions

DB and LN conceptualized the review. LN drafted the article, which was critically revised and edited by all co-authors. All co-authors approved the final version of the manuscript. All authors contributed to the article and approved the submitted version.
